# Promoting patient self-management following cardiac rehabilitation using a web-based application: A pilot study

**DOI:** 10.1177/20552076231211546

**Published:** 2023-11-08

**Authors:** Sabine Stamm-Balderjahn, Sebastian Bernert, Susanne Rossek

**Affiliations:** 1 Institute of Medical Sociology and Rehabilitation Science, 14903 Charité – Universitätsmedizin Berlin, Berlin, Germany

**Keywords:** Cardiovascular disease, cardiac rehabilitation, apps, self-monitoring, web-based applications

## Abstract

**Background:**

The use of health-related mobile apps has become an important component of healthcare. Patients can use a range of tools to strengthen their health literacy and promote disease management. The aim of the project was to develop a web-based application for use on smartphones, tablets and computers for patients with cardiovascular diseases (cardio-app).

**Methods:**

A semi-standardized written survey was conducted among rehabilitation patients with cardiovascular diseases (*n* = 158). The usability of the cardio-app was assessed using the System Usability Scale (SUS). The usage behaviour was conducted with a self-developed questionnaire.

**Results:**

The study enrolled 158 eligible rehabilitation patients. The SUS of the cardio-app determined was 74.4 (SD ± 17.4). For 86%, the menu navigation was self-explanatory and logical. The visual presentation appealed to 92% of respondents. The content of the texts used in the app was understandable for 95%, and 93% found the technical terms used in the glossary well explained. For 57%, the app was helpful in planning their physical activities. 83% of the rehabilitation patients would recommend the app to others. The main criticisms of the app were the lack of synchronization options with other apps. Of those who did not use the app, the following reasons for non-use were most frequently cited: too much effort (43%), lack of time (29%) and pandemic-related reasons (29%).

**Conclusions:**

The cardio-app revealed high agreement values. Whether the use of the app is associated with improved clinical state and outcome would have to be verified in further studies.

## Introduction

### Background

In Germany, most people are still dying from diseases of the circulatory system; in 2021, around 341,000 deaths were attributable to this. Of these, around 45,200 people died of a heart attack; men were affected significantly more frequently than women.^1^ This raises the need for action, also with regard to care processes.

In addition to the acute treatment of cardiological patients, a differentiated care structure is available in Germany, which serves the professional, domestic and social reintegration of those affected. Whether a rehabilitation measure should be carried out is already assessed in the acute hospital. In particular, for patients after acute coronary syndrome, participation in cardiac rehabilitation has been shown to be effective in reducing all-cause mortality.^2,3^ Here, the early start of a motivating and medical rehabilitation is of great importance. Accordingly, early initiation of rehabilitation improves mortality in patients after myocardial infarction.^4^ In a randomized controlled trial, Peixoto et al.^5^ showed improved health-related quality of life (HRQL) and functional capacity in patients who had recently experienced an acute myocardiaI infarction, when a program with progressive exercises started early. In patients after bypass surgery, prolonged onset of rehabilitation resulted in less progress in cardiovascular fitness, resting heart rate and body fat percentage.^6^ A significant reduction in mortality and rehospitalization rates in patients with heart failure was shown in a study by Cai et al.^7^

Within the diagnostic spectrum, more than 50% of rehabilitation patients have coronary heart disease, most of which was revascularized by catheter intervention.^8^ Accordingly, this patient group suffers from a chronic coronary artery disease for which efficient follow-up care is of fundamental importance in order to consolidate the successes achieved during the rehabilitation with regard to the reduction of cardiac risk factors.^9^

In order to consolidate the successes achieved, targeted follow-up programs are needed. Although these programs have shown positive effects, they often relate only to a part of the relevant cardiovascular risk factors and only to the rather medium-term observation period. Long-term evidence of effectiveness – observation period is longer than 1 year – is rare.^10^ Moreover, the programs are associated with high personnel, financial and time costs,^11^ which cannot be provided in routine care. An alternative may be to empower patients themselves to be autonomous managers of their health over the long term. The German Federal Association for Rehabilitation^12^ has proposed a follow-up passport as a means to transfer information to follow-up care providers and to motivate rehabilitants to continue with the lifestyle modification behaviours learned during rehabilitation. Such a follow-up passport for cardiac rehabilitation patients was developed in the project ‘Kardio-Pass’. It is used to document the course of therapy and care in the acute, rehabilitation and aftercare phases.^13^

A major potential for increasing the efficiency of this patient passport lies in its digital application. An example of the additional benefits compared with the paper version is that the courses for blood pressure and pulse can be recorded without any time limit. Furthermore, it is possible to keep an exercise diary over a very long period of time.

In principle, mobile apps offer the opportunity for patient participation and can support various phases of the care processes in healthcare.^14,15^ Preliminary studies have shown that especially patients with cardiovascular diseases can benefit from apps. Their development should be based on theoretical models explaining behavioural changes. Useful content includes game-like elements, reward systems and social media elements.^16^ The basis for developing our app was the HAPA (Health Action Process Approach) model, a social-cognitive process model of health action according to Schwarzer, 2011.^17^ The model defines two phases of action, the motivational and the volitional. In the latter, the aim is to start intended behaviour and maintain it over the long term. The app should support the continuation of the health behaviour learned during the rehabilitation phase. In particular, the app contains specific instructions for the successful implementation of physical activities (setting goals, planning action and monitoring success). The fact that health apps for monitoring symptoms in heart failure promote self-management, and engagement was recently shown in an integrative review.^18^

The use of health-related mobile apps will play an increasingly important role for elderly and chronically ill users.^19^ With the help of apps, affected individuals can use a range of tools that individually strengthen their health literacy and promote the development of resources for self-empowerment and disease management. On the one hand, this can be ensured by providing information about the disease and its risk factors, and on the other hand, it can be supported by the possibility of actively making health-related documentations independent of place and time.

Entries on physical activity, current medication, laboratory values and physiological parameters such as blood pressure and pulse can make a decisive contribution to self-management and healthier behaviour. As a supplement to the existing aftercare structures of medical rehabilitation, it is therefore conceivable to consolidate and continue the health skills taught there with the help of a digital application.^20,21^

While the acceptance of mobile applications is now well established, studies on their effectiveness are rather rare. The review by Payne et al.,^22^ for example, showed that smartphone apps improve physical activity behaviour, but the studies reported did not include patients with cardiovascular disease.

### Objectives

The aim of the project was to develop a mobile, cross-platform web application (web app) for use on smartphones, tablets and desktop PCs for patients suffering from coronary heart disease, heart failure, or arterial hypertension (cardio-app). A quantitative user and acceptance analysis followed the development. The construction of a purely mobile application was opposed by the fact that the target group to be addressed predominantly uses desktop PCs. When we started planning the cardio-app in 2016, 63% of 60–69-year-olds owned a desktop PC and 41% a smartphone.^23^ Cardiology patients participating in clinical trials have an average age of 61 years.^24^

The basis for the cardio-app was the cardio-pass developed in the research project ‘Kardio-Pass’ and tested by rehabilitation patients, which was designed for follow-up care and was available in paper form. The rehabilitees who had used the cardio-pass in this study rated it as a helpful tool for documenting follow-up data. However, a major disadvantage of the passport was the limited space for these entries. As a result, almost half of the respondents could imagine using such a passport in the form of a digital application in the future.^13^ First, the content of the cardio-pass was further developed based on the study results from the rehabilitation patient survey of this previous project. The result formed the template for the cardio-app to be developed.

The aim of the cardio-app should be to provide patients with cardiovascular diseases with a tool that enables them to bundle and store all important examination and therapy findings, from diagnosis to therapy to aftercare, in one central location. Patients should thus be able to obtain an overview for themselves of the treatments that have already been carried out and those that will be carried out in the future. Furthermore, it should be possible to present selected data – such as blood pressure values – to their attending physicians in the form of diagrams. For patients using the app with a PC, there should be the option to print out their collected data and present it to their doctor. The autonomous documentation of their own data and findings should help patients in managing and dealing with their illness more competently and actively. The cardio-app could thus help in not only supporting patients’ adherence to therapy but also promoting it.

The aim was to investigate the acceptance of the cardio-app and the frequency of use. In addition, how much the cardio-app was perceived as useful, supportive, motivating, and/or informative should be quantified. Qualitative and qualitative analysis procedures should be included in the evaluation.

## Methods

### Trial design and framework

The project was divided into two phases: a development phase and an evaluation phase. The development phase included the technical development of the cardio-app, involving the research institute and an external IT agency. In the evaluation phase, the cardio-app was tested by patients, and a written survey was conducted to assess its acceptance and usefulness. Results of this survey are reported below.

The basis for the development of the cardio-app was the paper passport created as part of the ‘Kardio-Pass’ research project.^13^ Almost the entire content of the passport was transferred to the app and adapted accordingly the results from the user and acceptance analysis of the previous study. During the development, the advantages that a digital version of the ‘Kardio-Pass’ offers compared to the paper form were primarily taken into account. These include the following: documentation of vital signs without space limitations and automatic display in a graph (data curve), more space for the medication schedule and the ability to export appointments to the individual calendar and upload documents to the app. As additional content, a glossary explaining important cardiology terms and extensive user instructions were developed. Furthermore, a digital reward system for the achievement of individually planned physical activities was created. When a goal was achieved, this was reported to the participants in the form of feedback messages. Thus, patients who achieved 50% of their weekly activity goal received the message: Very good! You are well on the way!

According to the professional code of conduct for physicians, every study involving human subjects must be submitted to the responsible ethics committee for review. The present study was reported to the responsible ethics committee of the Charité – Universitätsmedizin Berlin before the study was initiated. The study received a positive vote under application number EA4/028/19 on 9 April 2019.

The study has been registered with the German Clinical Trial Register (DRKS) under the registration number DRKS00019041.

### Technical development

**Figure 1.**
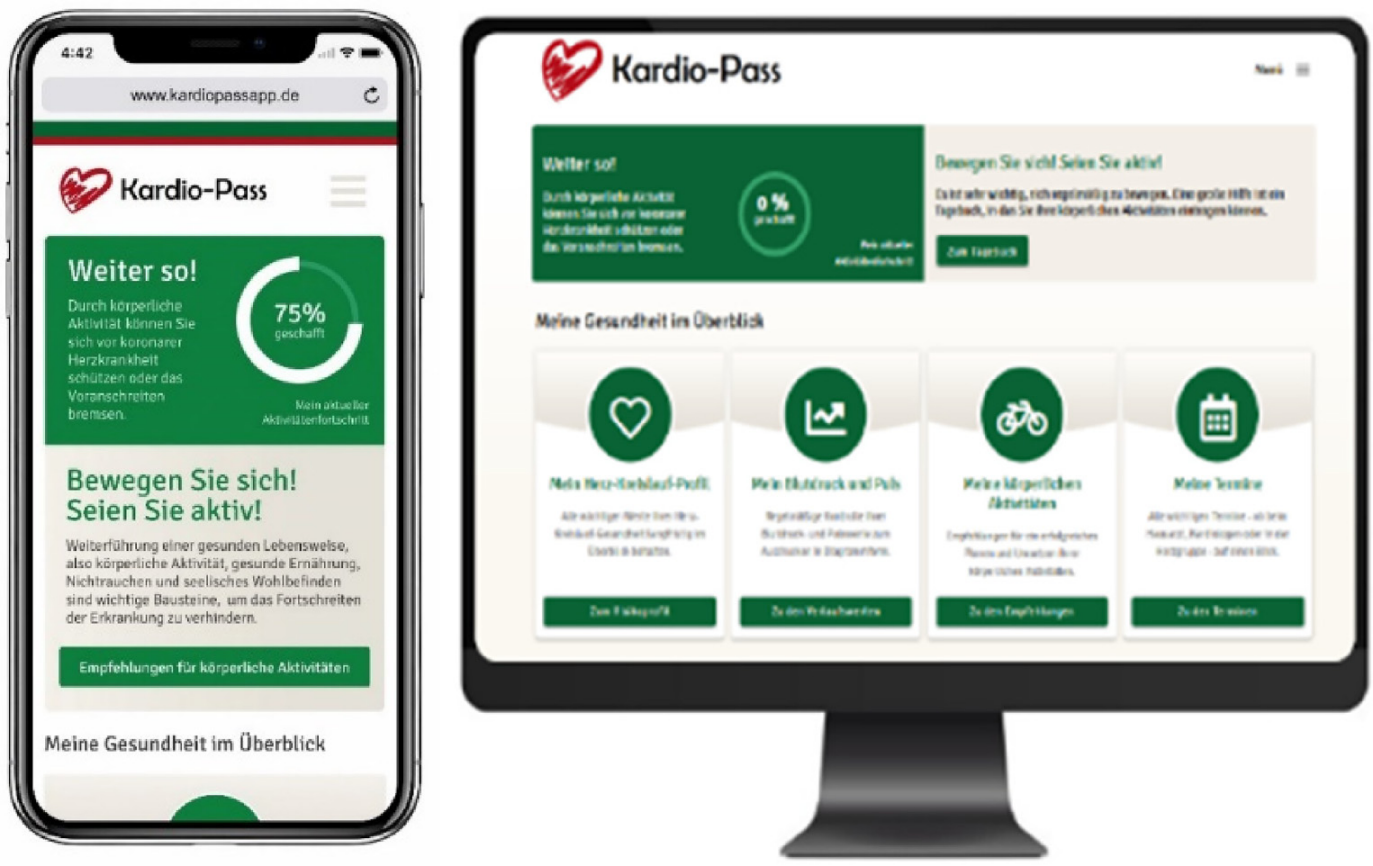
Screenshots of the cardio-app, assessed by smartphone and computer.

The first study phase consisted of the technical development of the cardio-app, which can be assessed by smartphones, tablets and computers. The views of the application on the smartphone and computer are shown in Figure 1.

An external IT agency was assigned for the development. First, the content, technical and design framework of the cardio-app was defined in a conceptual phase. Based on this, a wireframe concept was developed, which was further enhanced into a detailed UI/UX design. This formed the basis for a consistent and user-centric design grid for all page types, incorporating the frontend framework *Bootstrap* as a technological base.

In the implementation phase, a TYPO3 content management system (CMS) was set up in the current version. The technical development of the web layout was realized with the angular-based open source web framework *Ionic*, which was specifically designed for the creation of so-called hybrid apps. With the help of the agency's own TYPO3 extension T3REST, it was possible to connect *Ionic* with the TYPO3 entity. Thus, TYPO3 serves as backend, database and content management system for the study management to change static page content, such as impressum or contact, while *Ionic* provides both the design components and, in combination with T3REST, the dynamic page content.

The data entered by patients was stored in the TYPO3 backend and displayed as dynamic content in the frontend of the web application, within the patient's personal profile, using the TYPO3 extension T3REST. For reasons of data protection, the personal information entered could only be viewed by the patients or, with their permission, also by the attending physicians and could not be viewed via the TYPO3 backend. By using *Ionic* as the frontend component, the cardio-app could be displayed for any screen size and was thus independent of the type of end device used. In addition, it was particularly important to optimize the web app for as many web browsers as possible. Since the target group consisted in part of users who were at an advanced age, less widespread browsers had to be taken into account here, which are found as standard applications on diverse terminal devices and thus have a variety of specific usability requirements.

At the end of the technical development phase, extensive user tests were conducted. The tests were carried out on the basis of a task protocol that described quite specifically what was to be tested. The focus here was on the exploratory character. The aim was to check whether the app functioned properly or there were any malfunctions.

The test results provided suggestions for improvement; likewise numerous errors could be uncovered. The tests revealed that there were major shortcomings especially when using the cardio-app on mobile devices. The problems varied depending on the type of device or browser. For example, difficulties occurred primarily with one device model and its preinstalled browser. For example, entries that were properly entered in a table form on the edit page appeared, but did not appear in the correct column on the view page. Minor inadequacies, such as the absence of unit specifications, small spelling errors, inconsistencies between the headings within the app and the menu headings, could be identified and corrected. A correction of the described problems took place before the app was delivered to the study participants.

The cardio-app includes the menu items listed in Table 1. Patients can make entries in nine of the menu items.

**Table 1.** The menu items of the cardio-app.

Menu item	Content	Type of entries
Patient data	Relevant information in the event of an emergency	Existing allergies, anticoagulant medication is being taken
Diagnoses	Diagnoses	Cardiac and concomitant diagnoses. Medical reports can be inserted as PDF files
	Interventions	Date and type of interventions, i.e. stent implantation
	Echocardiography	Results of investigation
Medications	Intake regimen	Trade name, agent, form, dosage and timing of intake, reason and duration of use
Blood pressure and pulse	Data collection over time (tabular and graphically form)	Date of measurement, explanatory remarks
Cardiovascular profile	Data collection over time (tabular form)	Date and type of behavioural and risk parameters, i.e. body weight, waist circumference, smoking status, serum lipids, HbA1c
Exercises	Recommendations for regular physical activity. Small motivational messages are provided to reward the user while encouraging compliance	Planning and documentation of physical activity with weekly goal setting
Heart group participation	Documentation	Data entry for place, time and training frequencies
Doctors and institutions providing treatment	Address fields	Data entry for addresses and contacts
Appointments	Appointment management	Planning and documentation of medical consultations and course participation, i.e. smoking cessation
Signs of a heart attack	Schematic illustration of heart attack signs and information what to do in an emergency	-
Instructions for using the application	Short video sequences	-

### Participants and recruitment

The inclusion criteria were age 18–80 years, having a cardiovascular disease, owning a smartphone or having access to the internet via a computer or tablet, and sufficient knowledge to use them. Study inclusion was performed in six rehabilitation clinics by study coordinators (physicians, nurses). The cardio-app was presented in the facilities onsite either by the physician personally, a clinic staff member or a member of the research team. Prior to study inclusion, each patient was given the study information, the informed consent form and the personal access data for the cardio-app. A brief personal introduction to the use of the app followed, so that patients could make initial entries in the app (e.g. diagnosis and findings data) with the help of staff. Additional support in using the app was provided by the comprehensive user instructions and short video sequences that are part of the app. It was voluntary how often the patients used the app. A control of the entered values by the research team was not intended in this study.

Participation in the study was voluntary. Informed written consent was obtained from all patients when enrolling them in the study.

### Statistical methods

A semi-standardized written survey was conducted among rehabilitation patients with cardiovascular disease. The rehabilitants received a questionnaire after using the app for 5 months.

The questions on user-friendliness were asked on a 5-point Likert scale (‘strongly disagree’ to ‘strongly agree’) using the established instrument by Rummel,^25^ which is available in German. The remaining questions were self-developed. Closed questions were asked for each individual topic block (e.g. functionality of the app); they were measured with a 4-point scale: ‘strongly agree’, ‘agree’, ‘disagree’ or ‘strongly disagree’. These questions included an additional user-defined text field for feedback responses. A 5-point scale was used for the overall evaluation of the app (‘very good’ to ‘very poor’). Rehabilitees who did not use the cardio-app were asked about their reasons.

The statistical evaluation of the questions on usability (5-point Likert scale) was conducted using the ‘System Usability Scale’ (SUS) according to Brooke.^26^ The final score for the SUS can range from 0 to 100. Higher scores indicate better usability. The SUS has proven to be a reliable and valid instrument for measuring the usability of digital systems. As an example, the work of Orfanou et al.^27^ reported good internal consistency with a Cronbach's alpha of 0.82. The scale was also used in studies involving patients with cardiovascular disease.^28–31^

The specific answers to the questions regarding functionality, design/layout, information/content, usefulness, etc. (four-level response scale) were dichotomized into the categories ‘agree’ (‘strongly agree’ as well as ‘agree’) and ‘disagree’ (‘disagree’ as well as ‘strongly disagree’).

Data were analysed using descriptive statistics such as frequency distributions, mean comparisons and chi-square tests. Binary logistic regression analysis was used to test for differences in socio-demographic variables (sex, age, marital status, occupation and education) between users and non-users of the app. Linear and ordinal regression analyses were performed to test the relationship between SUS scores, overall rating of the app and usage behaviour with gender and a dichotomous age group (under 59 or 59 and over) and their interactions. The level of significance was assigned at *p* < 0.05. The statistical analysis was carried out using the SPSS statistics program, version 28.

Qualitative content analysis methods were applied to the responses in the user-defined text boxes. For evaluation, the answers to the questions in the categories of functionality, design/layout, information/content and usage behaviour were considered. In a first step, the respondents’ statements were reduced by identifying the core content. In the process, it was noticed that some answers did not fit the respective category, but could be assigned to another one. For example, in the information/content category, suggestions for improvements to functionality were mentioned. These were then assigned to the functionality category accordingly. In a second step, core content with identical statements was grouped together and listed within the respective category according to the frequency of mentions. Only statements that were mentioned more than once were included in the analysis.

## Results

### Quantitative analysis

**Figure 2.**
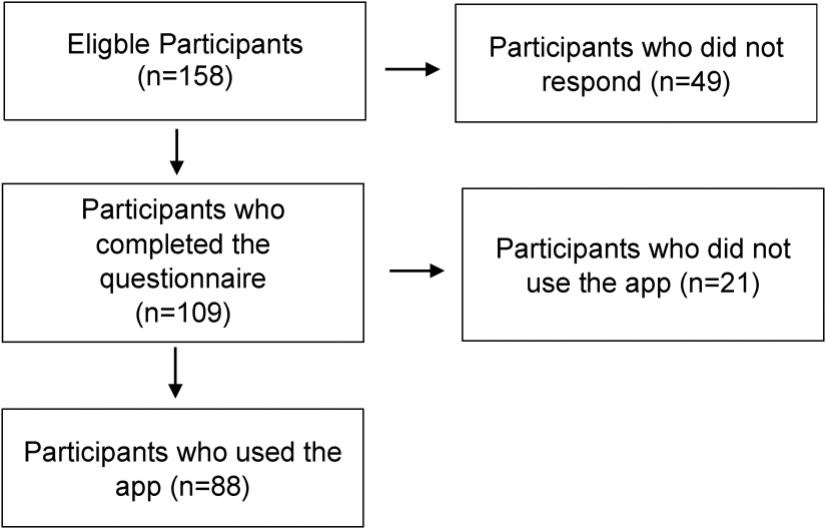
Flowchart of participation.

The study consisted of 158 eligible rehabilitees. A flowchart of participation in the different phases of the survey is shown in Figure 2. Their mean age was 57.9 years ± 10.9 (range: 19 to 80). Of those surveyed, 82.2% were male and 17.8% female. The completed questionnaire was returned by 109 of them, representing a response rate of 69%. Of the respondents, 65% were employed, of which 79.1% worked full time. Most of the rehabilitants (80.6%) lived in a partnership or family, while 19.4% lived alone. The majority had a high school diploma (49.5%), 39.8% had a middle school diploma and 10.7% a low one. The cardio-app was used by 80.7% (*n* = 88) of respondents. Of those who did not use the app (*n* = 21), information is available on the reasons for non-use.

**Figure 3.**
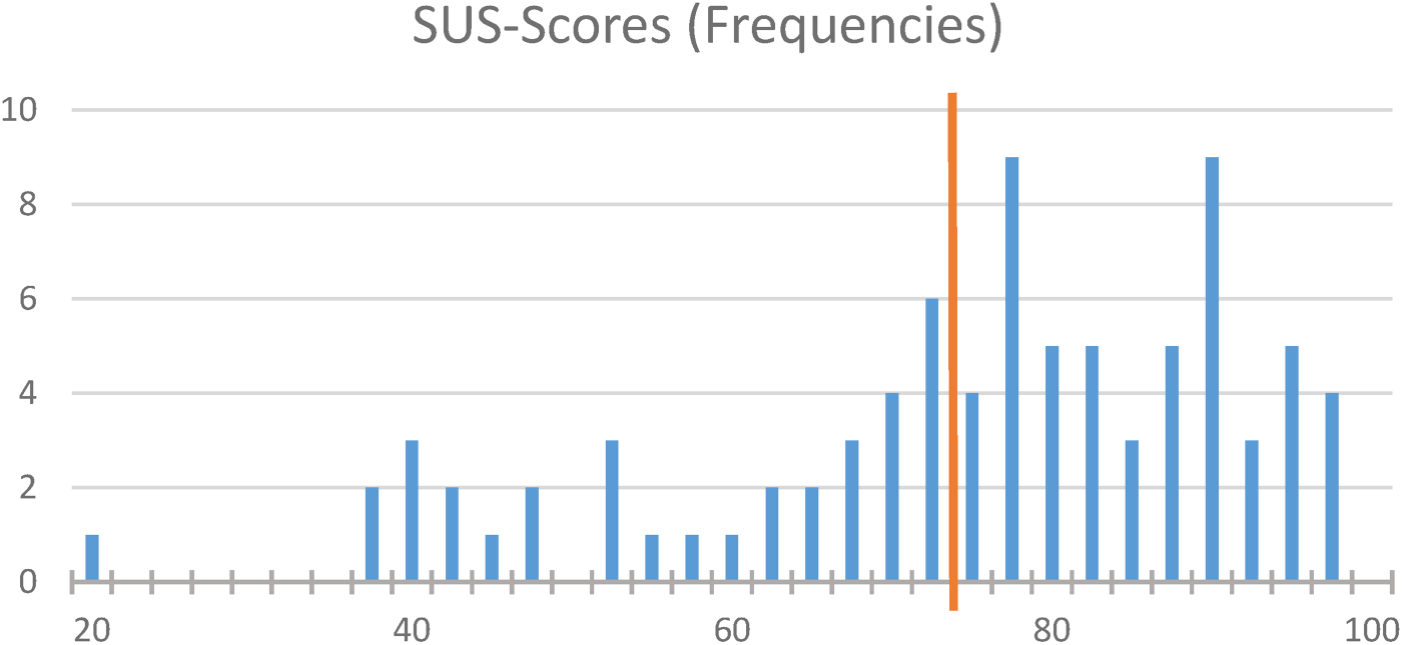
Frequency distribution of System Usability Scale (SUS) scores.

The usability score of the cardio-app obtained with the System Usability Scale (SUS) was 74.4 (SD ± 17.4), with a median of 77.5. Good usability is attested to a system with a mean value of 72.8 or higher.^32^ Figure 3 shows the distribution of the SUS scores displayed as histogram with a cut-off value of 72.8.

**Figure 4.**
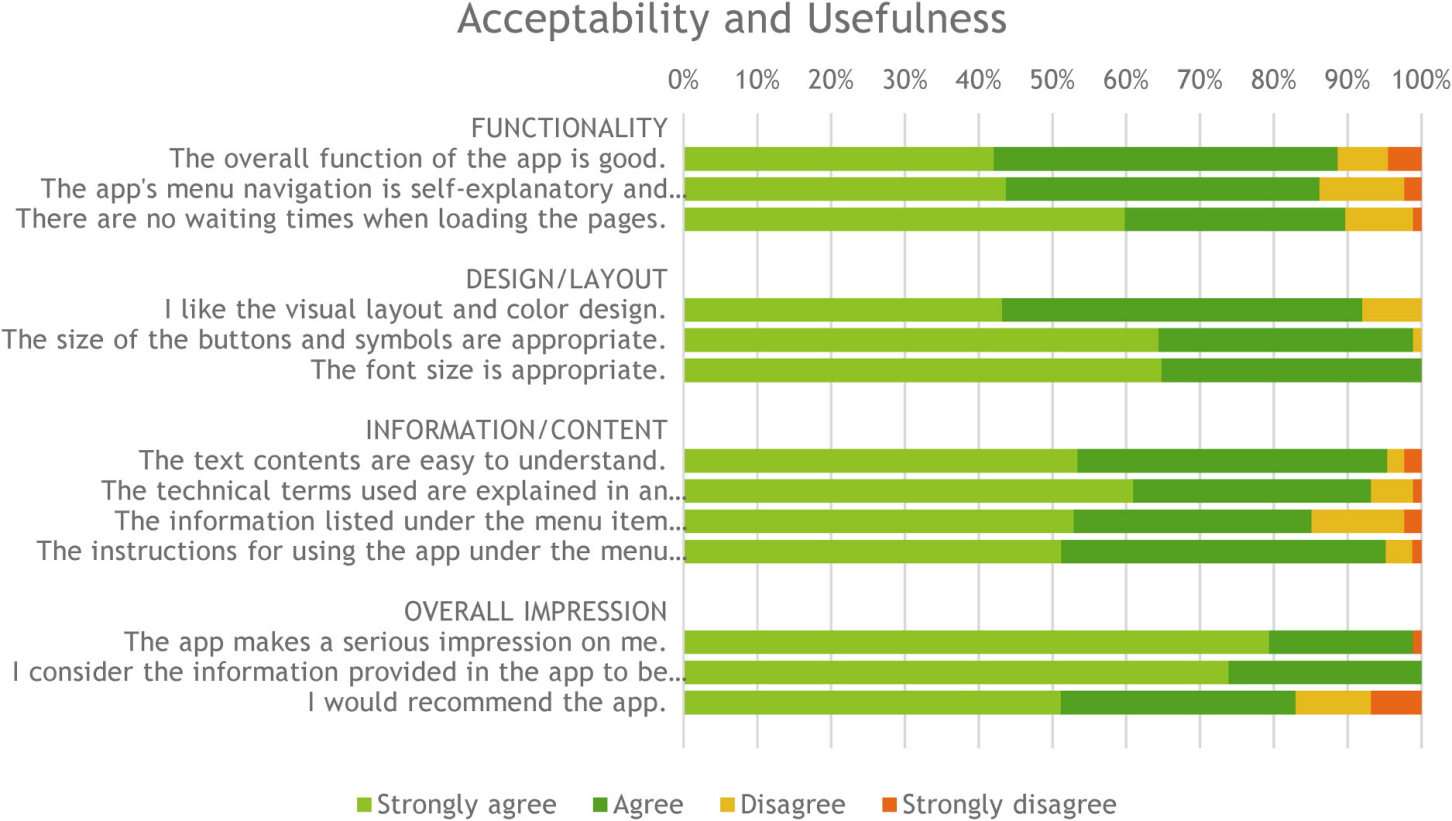
Ratings of functionality, design, content and overall impression of the app.

The ratings for the topics of functionality, design/layout, information/content and overall impression of the cardio-app are shown in Figure 4. The best rating (full agreement) was given to the statement ‘The app makes a serious impression on me’ with almost 80%.

The details of the dichotomized results concerning functionality, design/layout and information/content of the app showed that the menu navigation of the app was self-explanatory and logical for 86.2% of the rehabilitation patients. The visual layout and colour design appealed to 92% of the users. The content of the texts used in the app was understandable for 95.5%, and the technical terms used in the glossary were well explained by 93.1%. The information on aftercare options (e.g. IRENA program) was found useful by 85.1% of rehabilitants.

Further surveys were related to the categories of usage behaviour, usefulness, safety, motivation and technical features.

**Figure 5.**
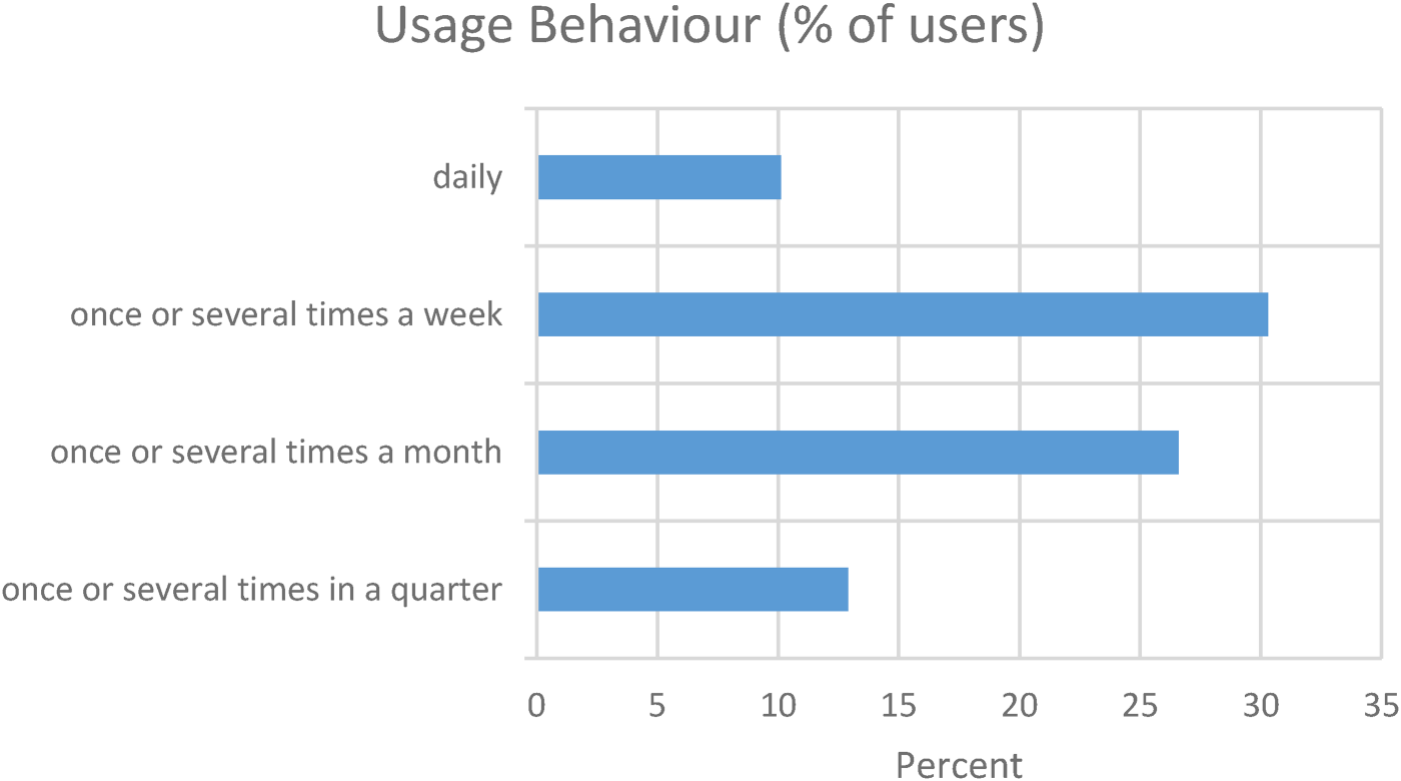
Usage behaviour percentages.

In the usage behaviour category, the frequency of the use of the cardio-app was surveyed. Here, the answer ‘several times a week’ (24.1%) was predominantly given, followed by ‘several times a month’ (20.7%), ‘once a week’ (13.8%) and ‘daily’ and ‘once a month’ (12.6% each). The app was used ‘once a quarter’ by 11.5% and ‘several times a quarter’ by 4.6% of respondents. Figure 5 shows how many people used the app during which time period. The answers were summarized in four categories.

In the usefulness category, three situations were asked in which the cardio-app was perceived as helpful (multiple answers were possible). The coordination of appointments with the help of the app was useful for 26.1% of the rehabilitation patients. It was helpful in communicating with physicians for 35.2% and in planning their physical activities for 56.8% of respondents. For 19.3% of the rehabilitants, the app was not useful.

In the category of safety, three situations were asked in which the app evoked a feeling of safety among users (multiple answers were possible). Safety was felt by 15.9% of users when they were out and about, and 21.6% felt safe using the app when taking medication. About half of users (45.5%) considered the app to be a crucial tool in the event of an emergency. The app did not convey a sense of security for 35.2% of respondents.

Two questions were related to motivational reasons for using the app. Being aware of regular check-ups motivated 39.8% of respondents to use the app. Being physically active on a regular basis was a motivation for 62.5% to use the app.

**Figure 6.**
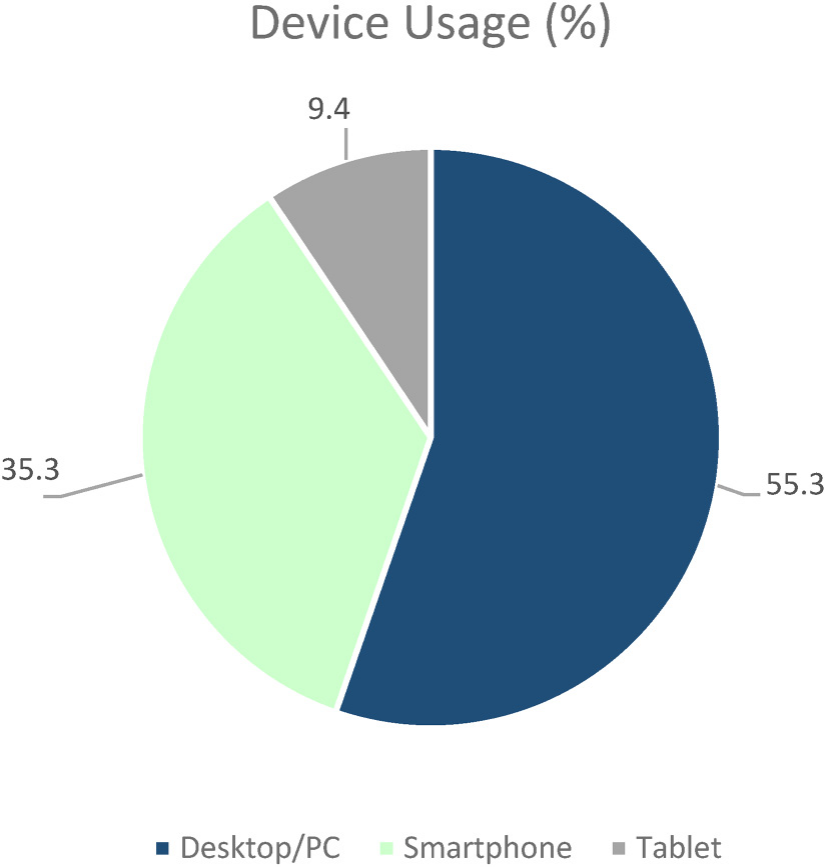
Device usage.

The most frequently used device with which the app was used was the computer, followed by the smartphone and tablet (see Figure 6). Thus, the app was most frequently used with fixed devices (55.3%). Logistic regression analysis revealed that older users (≥ 59 years) used the computer significantly more often than younger users (*p* = 0.05, odds ratio = 2.39).

The overall rating of the app (‘How do you rate the cardio-app overall’, Categories: very good = 5, good = 4, average = 3, bad = 2, very bad = 1) had a mean of 3.84, with a median of 4.0. Most (60.9%, *n* = 53) rated the app as ‘good’. 83% of the rehabilitants would recommend the app to others.

**Figure 7.**
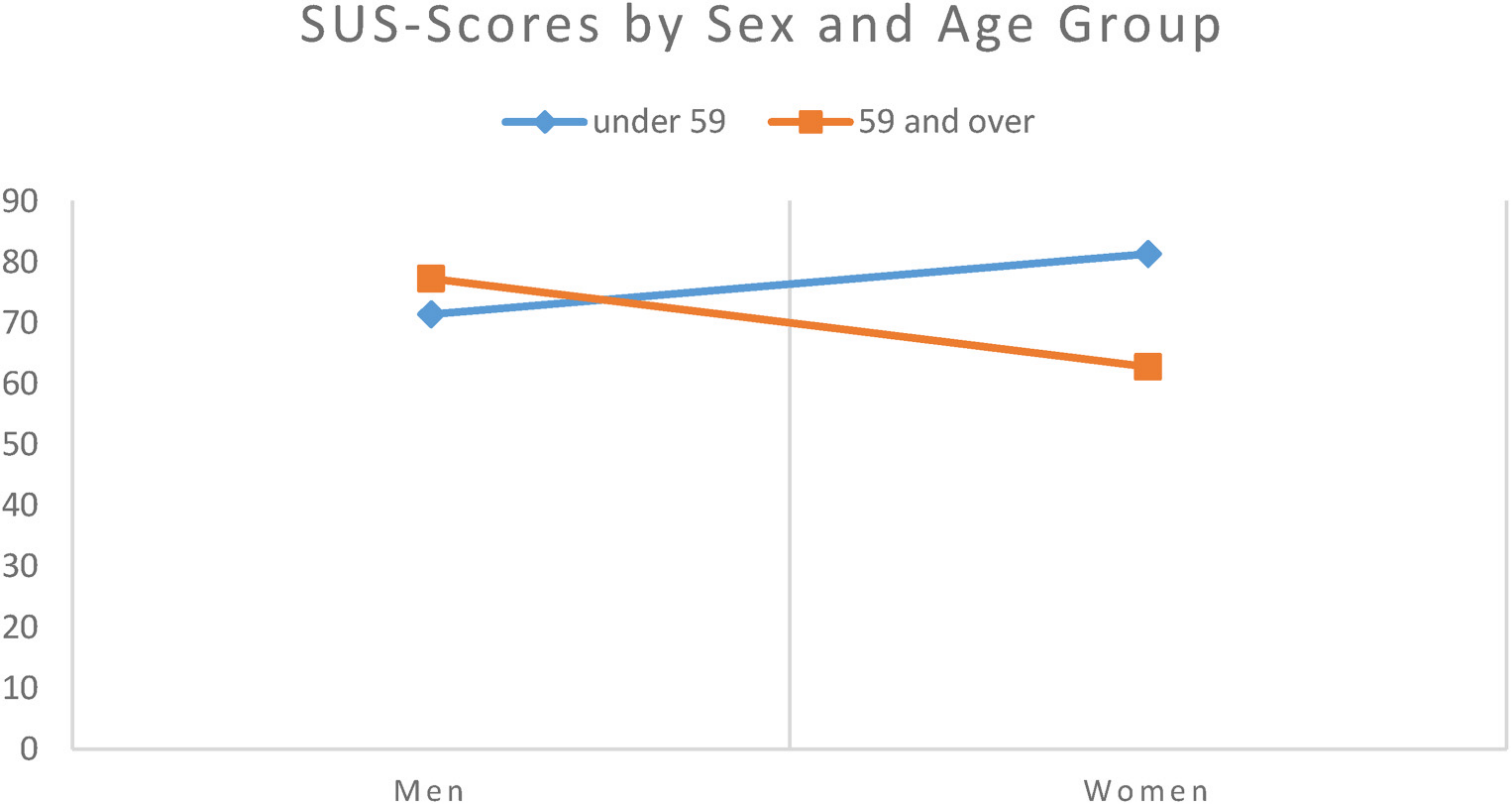
Mean SUS scores in men and women by age group.

A linear regression analysis of the SUS scores was performed using sex (male or female) and age group (under 59 vs. 59 and over) and their interaction as independent variables. The effects of sex (*p* = .103) and age group (*p* = .167) were not significant, but the interaction term showed a significant effect between age group and sex (*p* = .006). Men showed an increase in SUS scores in the older age group (71.43 vs. 77.3), while women showed lower SUS scores in the older age group (77.3 vs. 62.78) (Figure 7).

To test whether these significant interactions also occurred for usage patterns (daily to once per quarter) and overall rating of the app (very good to very bad), two ordinal logistic regression analyses were performed. Neither usage behaviour (sex *p* = .226, age group *p* = .545, interaction term *p* = .211) nor overall rating of the app showed significant effects (sex *p* = .321, age group *p* = .201, interaction term *p* = .297).

It was found that 19.3% (*n* = 21) of the rehabilitees had not used the app. The most frequently cited reason for not using the app (multiple answers were possible) was ‘The effort seemed too great’, cited by 42.9% of non-users. Equally important, at 28.6%, were the reasons ‘I didn't have the time’ and ‘External circumstances – especially the coronavirus pandemic – placed a heavy burden on my daily life’. Less than one-fifth stated that their health did not allow them to use it (19.0%), followed by lack of clarity about what to enter into the app (14.3%). Using the app seemed too complicated for 14.3% of respondents. It was also stated by 14.3% that there were technical problems (compatibility problems between software/browser/terminal device).

Differences between users and non-users were not significant with respect to sex, age, marital status, education and occupation.

### Qualitative analysis

The evaluation of the user-defined text fields provided detailed information on the app's weaknesses and strengths. Numerous suggestions for improvement were made in the area of functionality. Considering the multiple responses, there were a total of 47 suggestions for improvement. The majority (*n* = 17) would welcome it if the cardio-app had interfaces to make it easier to transfer data. Other suggestions for improvement included the ability to delete entries again, upload multiple documents and use drop-down menus. Eleven suggestions for improvement were related to the documentation of blood pressure and pulse values, the most frequently mentioned suggestions were the following: chronological sorting of the values (newest value at the beginning and not at the end of the table), documentation of the time and a field in which comments on the measurement can be entered. The aforementioned suggestions were implemented during the study period. Four respondents wished to expand the documentation options. Thus, two of the respondents saw it as advantageous if cross-indication data could also be recorded. Three further suggestions for improvement were made regarding appointment management; for example, the appointment calendar should have a reminder function (see Table 2).

**Table 2.** Analysis of the user-defined text fields with regard to an improvement of the functionality.

Functionality	
Improvement suggestions	Number
Presence of interfaces	17
Possibility to delete entries	4
Uploading multiple documents	3
Drop-down function	3
QR code/barcode use for medication entries and lab scans	2
Blood pressure and pulse values	11
Documentation options	4
Appointment management	3

Ten of the respondents reported technical problems, as a result of which the functionality of the app was no longer fully given. Some of the problems mentioned, e.g. login problems and data storage problems, were successfully resolved during the study phase. For some technical problems, the cause remained unclear.

Three rehabilitees explicitly rated the design/layout of the app positively. Three others criticized the display of the app on the smartphone (too small to view).

On the topic of information/content, nine respondents gave positive feedback. A total of seven respondents appreciated that they were able to get a good overview of their data with the help of the app. Two others were very satisfied with the information/content. Five of the respondents indicated problems in understanding the content. It was suggested, for example, that fewer Latin terms or a combination of technical term and German term be used.

In the usage behaviour category, reasons could be given if the app was used only once or less than once a month. Eleven reasons were given (multiple answers were possible), which are listed in Table 3. The most common reasons were lack of time, using other apps at the same time and using other apps.

**Table 3.** Analysis of the user-defined text fields on the reasons for low use.

Low usage	
Reasons	Number
Lack of time	5
Simultaneous use of other applications	5
Using other apps	5
Missing interface	3
Forgotten to make entries	3
Loss of motivation	3
Benefit is low or nonexistent	3
App is too complicated	3
Health problems	2
Stable health situation	2
Data entry effort was to high	2

Analysis of the user-defined text fields in response to the question about the reasons for not using the cardio-app showed that some rehabilitation patients felt that the effort involved was too high in relation to the benefits. It should also be noted that some users already use other types of apps to document their health-related data.

## Discussion

### Key findings

To test the acceptability and usefulness of the app, 158 rehabilitation patients with cardiovascular diseases were included in the study. The usability score of the cardio-app assessed with the SUS was 74.4 (SD ± 17.4).

The app achieved high approval ratings, especially in the categories of functionality, design/layout, information/content and overall impression. The non-users mainly criticized that entering the data was too time-consuming.

### Interpretation

The availability of mobile applications in the setting of cardiological rehabilitation has increased significantly in recent years. However, the apps evaluated within studies are mostly English-language and relate to so-called ‘home-based’ or ‘mobile’ rehabilitation or can be used as a supplement to a rehabilitation intervention.

Beatty et al.^30^ presented an app with similar comprehensive features as ours, such as goal setting, monitoring of physical activities and documenting vital signs that achieved comparable usability ratings (SUS score: 76). The score for the cardio-app we developed was 74.4 (SD ± 17.4). Good usability is attested to a system with a value of 72.8 or higher.^32^

Web-based applications accompanying the usual program of a rehabilitation measure can improve the cardiovascular risk profile. These features were therefore a key element of the cardio-app. A study by Widmer et al.^20^ and a meta-analysis by Indraratna et al.^21^ showed that the independent recording of health-related parameters such as weight, blood pressure and eating habits can have a positive influence on rehabilitation compared to patients who did not use an app in addition to standard rehabilitation. The evaluation of these aspects of the cardio-app (content and functionality) by the study participants showed very positive results.

The app we developed contains the features relevant for monitoring cardiovascular disease. It was designed specifically for follow-up care and refers to the German treatment guideline for the rehabilitation of patients with cardiovascular disease.^33^ The quality and functionality of such apps were examined in a recent study by Meddar et al.^34^ The review evaluated 3121 potentially relevant English-language mobile applications (called native apps) commercially available for Apple and Google platforms, with content related to the American Heart Association (AHA) guidelines for cardiac rehabilitation.^35^ Apps designed for mobile cardiac rehabilitation were assessed to determine whether they met the core components of the guidelines, such as daily weight monitoring, performing physical activities, low-salt diet, medication adherence, scheduling and attending medical appointments and monitoring symptoms of cardiac health. The quality of these apps was measured using the Mobile App Rating Scale (MARS),^36^ an app assessment tool most commonly used by medical experts for scientific evaluation of mobile health apps. Using 29 items on subjective and objective characteristics, the quality and functionality of these apps were measured. Of the nine apps that met the inclusion criteria for the study, the app ‘My Cardiac Coach’, developed by the AHA, achieved the best results. Whether the app we developed would meet these standards would need to be investigated in further studies.

Digital applications that are specifically used for the follow-up care of cardiovascular diseases (phase III rehabilitation) are still comparatively rare – especially in German-speaking countries. Internationally, the study situation is much better, although most applications are limited to the modification of individual risk factors. Specific behaviours to reduce risk factors, such as physical activity, can be supported through the use of digital applications, by tracking physical activity and reinforcing behaviour through push messages (feedback, motivational reinforcement). Studies by Park et al.^37^ and Lunde et al.^38^ showed an increase in physical activity in the intervention groups.

The app we developed contains a wide range of functions regarding cardiac risk and protection functions, but it is only used for documentation. Although reports of findings (doctor's letters, laboratory findings, etc.) can be uploaded, some functions are missing, which is mainly caused by the software architecture. Because the application was designed as a web app, a reminder of an appointment, for example, is only possible indirectly via an export function. This was also one of the criticisms raised in our rehabilitant survey. On the other hand, the usability of the app with fixed devices is an advantage. The assumption that already existed in the preliminary stages of development that older persons would rather use fixed devices was confirmed. In our study, 55.3% of the participants used a computer. This was used significantly more often by older individuals (≥ 59 years) compared to younger individuals (*p* = 0.05, odds ratio = 2.39). In the study of Hong et al.,^39^ as many as almost three-quarters of study participants used fixed devices, although the average age was 10 years higher than in our study (68 vs. 58).

Of the respondents, 19.3% (*n* = 21) indicated that the cardio-app was not used by them. The most common reason given by 42.9% for not using it was that the effort required to populate the app with data seemed too great. It is indeed a considerable effort to fill the app with data, but this is limited to the initial entry. During the initial entry, various basic data (e.g. information on allergies, intolerances, addresses of treating physicians and facilities) must first be stored in order to be able to use the app appropriately.

### Future prospects

The results of this study showed a good acceptance and usefulness of the tested cardio-app. On the other hand, various points of criticism were raised – particularly with regard to the lack of synchronization options with other apps. Ultimately, however, it will not be possible to develop an app that meets all requirements. In our app there was no feature to contact the non-users. This function would have to be set up in the further development of the app. A message could be initialized in case of prolonged non-use, including a reminder. To decrease the rate of non-users, it might be useful to target the motivation for using such an app. Rogers Protection Motivation Theory^40^ could be applied for this purpose. It assumes that positive health behaviour follows from two appraisal processes (threat and coping) of a certain risk behaviour (e.g. smoking). Furthermore, the good acceptance and usefulness of the tested app can be communicated, primarily by physicians. Measures taken by the public policy and educational programs for digitals skills should support implementation, when the cardio-app can achieve positive effects with respect to cardiac risk factors. This would have to be verified in an efficacy study.

### Limitations

A limitation of the study was the willingness of the rehabilitants to participate and the actual use of the app. Nevertheless, there were no differences between users and non-users in sex, age, marital status, education and occupation. Therefore, we assume that there is no fundamental bias in the survey of participants versus non-participants. As planned, the study conducted was able to test only part of the goals addressed by the app. Therefore, in subsequent studies, questions regarding therapy adherence and empowerment should be investigated. Before conducting such studies, it would be desirable to include further improvements in the app, such as the ability to synchronize with other health apps, which has not been possible so far due to lack of funding.

## Conclusion

The app showed a high level of user-friendliness. The menu navigation was simple and self-explanatory. Most users liked the design and the colour scheme. The content in the texts presented was comprehensible, and information on aftercare services was found useful. Weaknesses were found with regard to communication with doctors and the planning of sports activities. The main criticism was the lack of interfaces to other health apps to synchronize information and the limited possibility to upload documents to the app. Despite these weaknesses, the app was recommended by the majority of users.
